# Draft Genome Sequence of the Quorum-Sensing and Biofilm-Producing Pseudomonas aeruginosa Strain Pae221, Belonging to the Epidemic High-Risk Clone Sequence Type 274

**DOI:** 10.1128/genomeA.01343-14

**Published:** 2015-01-15

**Authors:** Alain A. Ocampo-Sosa, Marta Fernández-Martínez, Gabriel Cabot, Carmen Peña, Fé Tubau, Antonio Oliver, Luis Martínez-Martínez

**Affiliations:** a Servicio de Microbiología, Hospital Universitario Marqués de Valdecilla-Instituto de Investigación Sanitaria Marqués de Valdecilla (IDIVAL), Santander, Spain; b Servicio de Microbiología, Unidad de Investigación, Hospital Universitario Son Espases, Instituto de Investigación Sanitaria de Palma (IdISPa), Palma de Mallorca, Spain; c Servicios de Enfermedades Infecciosas y Microbiología, Hospital Universitario de Bellvitge-IDIBELL, Barcelona, Spain; d Departamento de Biología Molecular, Universidad de Cantabria, Santander, Spain

## Abstract

Pseudomonas aeruginosa Pae221 is a clinical isolate from blood culture. Pae221 was found to be a strong quorum-sensing and biofilm-producing strain and also demonstrates a notable production of phenazines. This strain belongs to sequence type 274 (ST274), an epidemic high-risk clone. Here, we report the draft genome sequence of P. aeruginosa Pae221.

## GENOME ANNOUNCEMENT

Pseudomonas aeruginosa is a ubiquitous Gram-negative bacterium whose metabolic versatility allows it to thrive in diverse ecological niches. In the human body, P. aeruginosa is able to transform into a hostile opportunistic pathogen, mainly when there is a breach of host tissue barriers or a suppressed immune system (1).

The successful combination of a number of intrinsic and acquired resistance mechanisms as well as the expression of numerous secreted virulence factors make this bacterium a threatening human pathogen. The expression of virulence genes in P. aeruginosa is found to be modulated by a bacterial cell-to-cell signaling mechanism, widely known as quorum sensing (QS) (2). QS bacteria produce and release small signaling molecules that at a high population density interact with associated receptors to induce the expression of various genes, including those related to biofilm production and virulence (2).

Here, we present the draft whole-genome sequence of P. aeruginosa Pae221, a QS and biofilm-producing strain isolated from a patient with bacteremia during a multicenter study, which included 10 Spanish hospitals (3). Pae221 also presents an enhanced production of phenazines when cultured in medium supplemented with divalent cations. Pae221 belongs to sequence type 274 (ST274), an epidemic high-risk clone circulating in Spain, which is also found in other European countries and Australia (4–8).

The P. aeruginosa Pae221 genome (Pae221_ST274) was sequenced using an Illumina HiSeq 2000 genome sequencer (with 100-bp paired-end reads) (GATC Biotech, Konstanz, Germany). The total number of paired reads obtained was 7,346,760, providing approximately 226-fold coverage of the genome. The sequences obtained were used for *de novo* assembly using SPAdes version 3.1.0 (9). The quality of the genome assembly was assessed in QUAST (10). The draft genome sequence consists of 353 contigs, with an *N*_50_ contig size of 339.356 nucleotides and a total length of 6,364,167 bp; the G+C content is 66.3%, similar to that of other P. aeruginosa sequenced genomes. The assemblies obtained were annotated using the Era7 tool BG7 (11). A total of 5,761 coding sequences (CDS) and 67 tRNA genes were identified in Pae221. Using the Harvest alignment tool (12), we determined that the most similar genome to Pae221 (91%) was that of the Liverpool epidemic strain LESB65, a QS overproducer hypervirulent strain (13). Pae221 possesses 40 CDS that are not present in the LESB65 genome. Among these, 4 genes were related to phenazine biosynthesis, similar to those described in P. aeruginosa isolate ATCC 700888 (14). More detailed analyses of the Pae221 strain, including comparative studies with other P. aeruginosa strains and transcriptome analysis, are in progress.

### Nucleotide sequence accession numbers.

The draft genome sequence of P. aeruginosa Pae221 has been deposited in GenBank/ENA under the accession no. CDFS01000000. The version described in this paper is CDFS01000001.1 and consists of the contig sequences CDFS01000001 to CDFS01000370.
